# The Effect of PRP Augmentation of Arthroscopic Repairs of Shoulder Rotator Cuff Tears on Postoperative Clinical Scores and Retear Rates: A Systematic Review and Meta-Analysis

**DOI:** 10.3390/jcm12020581

**Published:** 2023-01-11

**Authors:** Ioannis Angelos Trantos, Elias S. Vasiliadis, Filippos S. Giannoulis, Eleni Pappa, Fotios Kakridonis, Spyros G. Pneumaticos

**Affiliations:** 1 5th Department of Orthopaedic Surgery, KAT Hospital, 14561 Athens, Greece; 2 3rd Department of Orthopaedic Surgery, KAT Hospital, University of Athens, 14561 Athens, Greece; 3 Hand, Upper Extremity and Microsurgery Department, KAT Hospital, 14561 Athens, Greece

**Keywords:** arthroscopy, growth factors, platelet-rich plasma, platelet-rich fibrin, rotator cuff tears, shoulder injuries

## Abstract

The aim of this review and meta-analysis is to assess recent clinical trials concerning the combination of operative treatment of rotator cuff tears and the administration of PRP and its effect on clinical scores and postoperative retear rates. The trials were used to compare the combination of PRP treatment and arthroscopic rotator cuff repair to arthroscopy alone. Twenty-five clinical trials were reviewed. A risk-of-bias assessment was made for all randomized clinical trials included, using the Cochrane collaboration’s tool as well as a quality assessment for all non-randomized studies utilizing the Newcastle–Ottawa scale. The PRP-treated patients showed statistically significant improvement postoperatively compared to control groups concerning the Constant–Murley (mean difference 2.46, 95% CI 1.4–3.52, *p* < 0.00001), SST (mean difference 0.32, 95% CI 0.02–0.63, *p* = 0.04), and UCLA (mean difference 0.82, 95% CI 0.23–1.43, *p* = 0.07) scores. A statistically significant decrease of retear rates in the PRP-treated patients, with a risk ratio of 0.78 (95% CI 0.65–0.94, *p* = 0.01), was found. We believe that the results presented have positive aspects, especially concerning the retear risk, but are yet inconclusive concerning clinical results such as shoulder pain and function.

## 1. Introduction

Rotator cuff tears are among the most common causes of shoulder pain and disability treated by orthopedic surgeons worldwide, as it has been estimated that about 30% of patients older than 60 years of age have some form of rotator cuff injury [1]. Although surgical repair of such tears is considered the standard treatment, it has alarmingly high rates of retears and recurrent symptoms. Postoperative retear rates vary and have been reported to be up to 70% [2] and even 96% for massive tears [3]. Patients with structural failure commonly experience significant pain relief, but they often complain of weakness and have functional impairment of varying degrees [4].

Surgical failure may be influenced by a range of factors including patient age, body mass, other systemic diseases, smoking and physical activity history, tear size, tear type, tear location and surgical technique employed, tendon quality, fatty infiltration, degree of tendon retraction and chronicity of the tear, previous and/or concomitant surgery, and postoperative rehabilitation [5]. It is necessary, in order to decrease failure rates, since most of those factors cannot be modified by the surgeon, to improve any “weak links” of the current standard treatment. Concerning the cause of a possible retear, it has been demonstrated by biomechanical studies that the modern suture constructs can withstand loads that exceed physiological values of the rotator cuff tendons [6,7,8]. It has been indicated that the biomechanically “weak link” is the poor healing potential of the tendon, as scar tissue is produced, which has lesser ability to withstand tear forces compared to normal, healthy tendon tissue [9]. As has been stated by Castricini [10], the rotator cuff has limited ability of healing at its insertion on the humerus after repair, possibly not only because of the poor vascularization of tendon tissue but also because of the histopathologic changes that accompany a rupture (such as the high number of metalloproteases and their inhibitors, TIMP-1 and TIMP-2 [11]).

The research and clinical application of biological systems that can support the repair mechanisms of the tendon acquires an important meaning, in particular, the contribution of growth factors. PRP is an autologous blood product that contains an abundance of growth factors and bioactive cytokines, including vascular endothelial growth factor, insulin-like growth factor, fibroblast growth factor, platelet-derived growth factor, transforming growth factor β, and epidermal growth factor [12]. These bioactive cytokines may reduce inflammation and promote healing by augmenting cellular migration, cellular proliferation, angiogenesis, and matrix deposition [13].

The specific process of producing a platelet-rich plasma mixture may vary and has not yet been fully standardized. The variations of parameters, such as the different preparations kits and centrifuge process (often at “the point of care”), the blood harvested and its preservation during the process, the concentration of fibrinogen, and the fibrin polymerization process can lead to a spectrum of PRP products, which have different texture and concentration of cells and biologic molecules and may lead to different treatment results [14]. PRP products can be classified as either pure (or leucocyte-poor) or leucocyte- rich according to their leucocyte concentration, as liquid “plasma” or gel-like “fibrin, according to their texture and are used in activated form when combined with autologous thrombin and/or calcium, or in nonactivated form.

The application of PRP to promote tendon healing has been investigated, among other areas, to treat tendinopathy of the patellar, Achilles, and lateral elbow tendon [15]. However, concerning the treatment of rotator cuff tears, scientific consensus has not been achieved concerning the clinical application of PRP, and such treatment is not included in the guidelines of the American Academy of Orthopaedic Surgeons [16]. It also has to be noted that the centrifugation process of PRP production may concentrate potentially deleterious agents as well, and even among the potentially beneficial growth factors and cytokines, effects are often pleiotropic [17]. More research is necessary in order to understand how clinical outcomes are affected by participant characteristics or how the PRP formulation affects efficacy [18].

The purpose of this study is to review the current literature concerning clinical trials, which specifically assess the combination of arthroscopic surgical therapy and PRP administration for the treatment of shoulder rotator cuff tears. The primary question of this review is whether there is a significant improvement of the clinical results of the patients undergoing arthroscopic repair for rotator cuff tear combined with PRP treatment compared with patients undergoing the standard arthroscopic repair. The secondary question is whether the risk of retear is different between the above-mentioned patient groups. Our hypothesis was that PRP treatment would lead to better results concerning both of the above-mentioned questions.

## 2. Materials and Methods

### 2.1. Inclusion Criteria and Study Design

Full-length, English-language articles that reported clinical outcomes were screened for inclusion, dated from 2010 to the end of August of 2021. We decided to include any clinical trials that compared the combination of arthroscopic treatment for rotator cuff tears and administration of PRP products to arthroscopic repair alone. The time period between PRP administration and surgery should not have been longer than one month. The postoperative rehabilitation protocols must have been identical for the two patient groups of each study. The articles had to provide patient results measured in specific clinical scores and/or data of retear rates for a minimum postoperative period of six months. The clinical scores reported had to be any of the following: Constant–Murley, the American Shoulder and Elbow Surgeons Shoulder (ASES) score, the UCLA score, and the simple shoulder test (SST) since these were the scores most used in the literature. In addition, any articles concerning revision operations and studies where the patients have had previous operations on the affected shoulder were excluded as well.

### 2.2. Data Extraction

**Figure 1:**
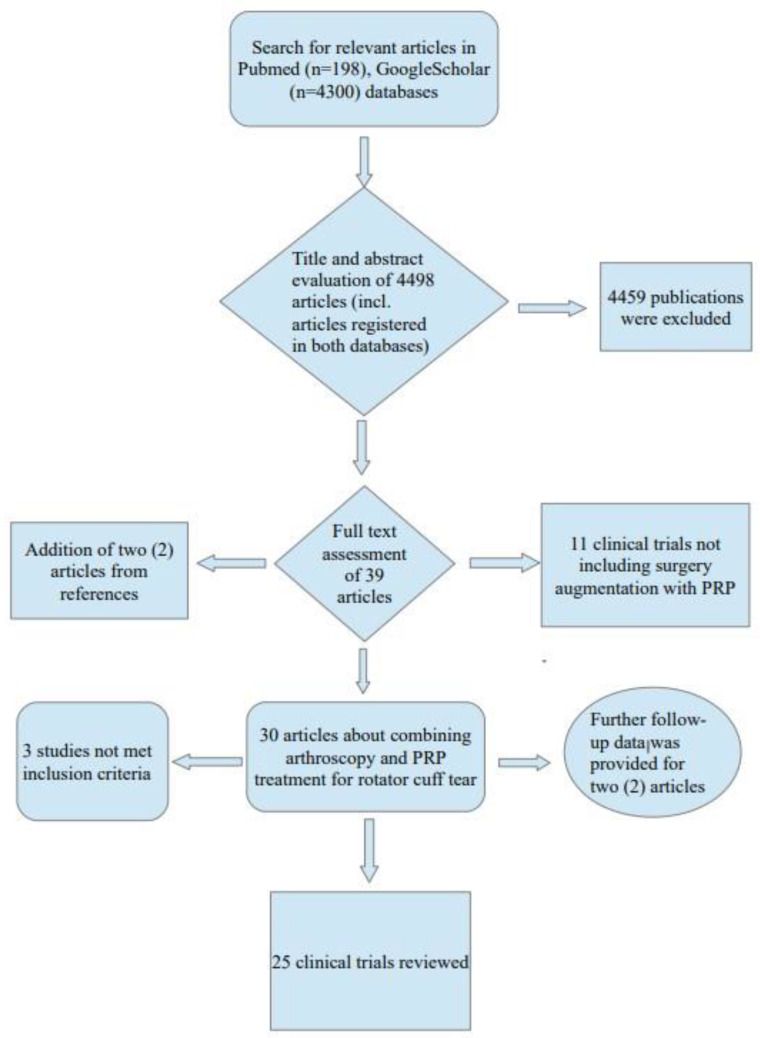
Flowchart.

The search was performed through the *PubMed* and *Google Scholar* databases. The keywords and phrases used were “shoulder rotator cuff tear”, “PRP”, and “arthroscopy”. The search provided 4498 publications, including articles found in both databases. Thorough assessment of the articles was performed through search engine filters, as the search was further restricted in articles that included at least two of the keywords or -phrases in their title, were classified as clinical trials, and published during the time period described in the inclusion criteria. The remaining articles were screened by the authors after evaluating the titles and abstract texts available. Based on the above-mentioned criteria, 39 articles were chosen for a full-text assessment, and 28 articles referring to 26 different clinical trials [5,10,19,20,21,22,23,24,25,26,27,28,29,30,31,32,33,34,35,36,37,38,39,40,41,42,43,44] were identified. In addition, through references in the articles chosen, two more published clinical trials were identified [45,46], adding to a total of 30 articles referring to 28 different clinical trials. The flowchart summarizing the above search procedure is presented in Figure 1. After assessment of the full text of articles, one study was excluded [35] because of lack of a control group of patients reported in the study, while two studies [22,29] were finally excluded because they did not use any of the clinical scores investigated and provided no data on postoperative rotator cuff retears.

### 2.3. Quality and Risk of Bias Assessment

Out of the 25 trials assessed, 16 were randomized controlled clinical trials. We used the Cochrane collaboration’s tool for assessing risk of bias in randomized trials [47] for these articles, and the results are summarized in Table 1. All but one of the trials provided information concerning the random sequence generation during the randomization procedure, while six of the trials lacked information of the allocation concealment. In a total of six studies, the blinding of patients, physicians, and/or the personnel assessing the outcome was inadequate, and in one study, the information concerning the blinding of physicians was lacking. Finally, one study was characterized as having an unclear risk of bias from other sources since it was completed earlier than originally planned because of the results of the interim statistical analysis.

As far as the non-randomized studies are concerned, we used the Newcastle–Ottawa Scale (NOS) for assessing the quality of nonrandomized studies in meta-analyses [48]. Concerning the assessment criteria, we considered a follow-up period of at least one year concerning the clinical scores and an imaging assessment for possible retears of at least six months postoperatively as adequate [49]. In addition, we accepted as adequate a follow-up rate of at least 90% about the clinical scores and 75% about the postoperative imaging. All articles had a rating of at least seven “stars” out of a possible nine in total. The quality assessment is summarized in Table 2.

### 2.4. Statistical Analysis

After identifying the clinical trials available, data concerning the number of patients included, the surgical techniques utilized, the PRP products used, as well as postoperative imaging studies and clinical follow-up were evaluated. The data were processed using the Review Manager (RevMan) computer program (version 5.4, the Cochrane collaboration, 2020). The trials were assessed for statistical differences of the clinical scores between control and treatment groups both preoperatively and at the final follow-up. The clinical scores chosen were those most commonly used among the trials reviewed, specifically Constant–Murley (for which 16 studies provided preoperative and 18 studies postoperative results), UCLA (7 studies preoperative, 8 studies postoperative results), ASES (7 studies preoperative, 8 studies postoperative results), and SST (6 studies). For one study [40], the standard deviation of the postoperative Constant–Murley and ASES scores was not provided and was calculated based on the confidence interval provided. The statistical method used was the mean difference method, with a confidence interval of 95%. In addition, the retear rates between control and PRP-treated groups of 25 studies were compared with the risk ratio method and a confidence interval of 95%.

## 3. Results

### 3.1. Arthroscopic Repair Techniques and Tear Characteristics

Concerning the trials reviewed in this article, in nine of them, the single-row technique was utilized, and in twelve, the double-row technique was used. Concerning the trials presenting double-row repairs, in four trials, three of which were published by Jo et al. [30,31,32], the suture bridge technique was presented, while in another, the knotless tape bridging technique was chosen [25]. Lastly, in four trials, either various techniques were used, or the specific technique preferred was undefined [19,21,39,40]. It has to be noted that all but one study mention the recruitment of patients with full-thickness tears, with the exception of the study of Weber et al. [42], which does not clearly specify whether patients with partial-thickness tears were included. The size of the tears of the patients included is not specified in fourteen of the studies, and the rest of the studies vary concerning the size of the tears from small tears (<1 cm in length) restricted to one tendon to massive tears affecting multiple tendons.

### 3.2. The Issue of Varying PRP Products

Concerning the texture of the product used, 12 trials used a liquid product, while 11 of them used a product with a gel-like texture, described as a fibrin matrix in several of these articles. In addition, in one trial, by Gumina et al. [27], the product was solid but formed as a membrane, which was intraoperatively applied at the tendon repair site, while the PRP texture and composition were not defined in one article. More specifically, six trials used pure PRP, six trials leucocyte-rich PRP, ten trials pure platelet rich fibrin (PRF), and two trials leucocyte-rich PRF. Unfortunately, few of the articles reviewed provided data concerning the specific concentration of cells and growth factors of the products administered. The trial by Pandey et al. [37] and the three trials by Jo et al. [30,31,32] reported mean concentrations of platelets, leucocytes, and red blood cells. Apart from those and the trial by Gumina et al. [27], which reported similar data for the PRF administered and collected by samples before the start of the trial, only rough estimates of the concentrations of the products used can be made based on the preparation process that has been followed.

### 3.3. Administration Time and Dosage Scheme

Most of the trials (22 of the 25 trials reviewed) proposed a single-dose treatment, with the product administered during the operation, after the tendon repair, and before wound closure through the arthroscopic portals. At one trial, a second dose was added postoperatively after one week [28]. One trial studied the administration of a single dose 10–14 days postoperatively [40], and one trial evaluated the administration of two PRP doses at one and two weeks after the surgical procedure [41].

### 3.4. Postoperative and Rehabilitation Protocols and Follow-Up Period

In general, no important differences concerning the postoperative treatment and the rehabilitation plans were found between the articles reviewed. Unfortunately, three of the articles reviewed [42,43,46] did not provide any data concerning the postoperative rehabilitation protocol, while the article by Antuna et al. [19] provided information concerning the shoulder immobilization period alone. All of the trials reported an immobilization period with a duration of 3 to 6 weeks immediately postoperatively. Usually, the arm was immobilized with a sling or an abduction pillow/brace, while one trial [45] reported the utilization of a Velpeau immobilizer. It has to be noted that 15 trials reported that passive motion, even if restricted, was allowed as soon as the first postoperative day to the tenth postoperative day, while 7 trials reported that no movement was allowed for the first 3 to 6 weeks. After this period, active motion (even if assisted) began at 3 to 6 weeks postoperatively for all the articles that reported such data. Muscle strengthening would begin as soon as six weeks postoperatively although in the majority of the trials, it started three months postoperatively along with light sport activities. Return to full activity was delayed until six to nine months postoperatively, and the follow-up period varied from 6 months to 5 years. It should be noted that most of the trials (eighteen of the trials reviewed) reported a mean follow-up period between one and two years postoperatively.

### 3.5. Clinical Results

All but one trial reported data concerning the postoperative clinical results of the patients, while one trial [46] reported data concerning retear rates but no comparable clinical scores. Concerning the trials reviewed, most of them (16 of 24 trials) showed no statistically significant difference between the patients treated with arthroscopic repair with the addition of PRP and the patients treated with the standard operative and rehabilitation protocols. Eight trials showed a statistically significant difference in certain aspects reviewed, including the trial by Ebert, Wang et al. [5,41], which did not show any significant differences at the initial published article but observed a 3.3-point higher Constant strength subscale score in the PRP group compared with the control group after adjusting for sex after 3.5 years of follow-up. No trial reported worse clinical parameters for the PRP-treated patients of any statistical significance.

Concerning the results reported, sufficient data to compare postoperative Constant–Murley scores between treatment and control groups were provided in 18 studies. After calculating the mean difference between these scores, it was found that the PRP-treated patients had higher scores, and this difference was statistically significant, while there was no statistically significant difference in the baseline scores reported (16 studies). However, this difference has been estimated to be quite low: of approximately 2.46 points (*p* < 0.00001) on the Constant scale. The results are similar concerning the UCLA score and the SST, with a statistically significant improvement in the PRP group of 0.83 points (*p* = 0.007, 8 studies) in the UCLA score and 0.32 points (*p* = 0.04, 6 studies) in the SST. In addition, processing the data concerning the ASES results did not result in any difference of statistical significance. It is worth noting that the differences in these results are of doubtful clinical impact, as they are lower than the minimal clinically important difference (MCID) for rotator cuff tear, which has been estimated to be between 6.7 and 26.9 points for the Constant–Murley score [50,51,52], between 2 and 3 points for the UCLA score [52,53], and between 1.2 and 4.3 points for the SST [54]. These results are presented in Table 3, Table 4, Table 5 and Table 6.

### 3.6. Retear Rates and Imaging Results

An important factor under survey was the postoperative structural integrity of the repaired tendon. All the studies examined postoperative imaging of the repair site. More specifically, fifteen studies used postoperative MRI, four ultrasound imaging, three MRI arthrogram, and three utilized a combination of imaging techniques, including CT arthrogram and simple radiographs, apart from those mentioned above. Most of the studies defined the imaging of full-thickness tendon defects at the repair site postoperatively as a “retear”. This definition refers to Sugaya grade IV and V in MRI imaging (grade I, sufficient thickness with homogenously low intensity; grade II, sufficient thickness with partial high intensity; grade III, insufficient thickness without discontinuity (thinned cuff); grade IV, presence of minor discontinuity; grade V, presence of a major discontinuity) [55]. Fourteen studies either provided specific data for the Sugaya classification of postoperative MRI images or clearly characterized rotator cuff status as a “retear” according to this classification. It is also notable that the study by Pandey et al. [37], which utilized ultrasound imaging, attempted to classify the imaging results to grades that are specifically described as similar to Sugaya grades. Therefore, it was decided to assign any patients with MRI imaging of Sugaya grade I–III to the “healed” patient group, whereas any patients with Sugaya grade IV–V were assigned to the “retear” patient groups during the statistical analysis. Only the article by Dukan et al. [25] characterized Sugaya grade III–V as retears but did not specify the exact grade for the patients in the study. Out of the 25 studies that compared imaging results of PRP and control groups, 7 showed a statistically significant decrease of the retear rates, while only 1 study showed a significant increase of the retear rate for the PRP group. It also must be noted that the majority of the rest of the studies showed improvement, although not significant, of the retear rates. This fact indicates that more powerful studies with larger study groups may provide stabler evidence concerning the trend of smaller retear rates after PRP administration.

The data provided by the above-mentioned studies were processed, and the results are showcased in Table 7. It is shown that there was a statistically significant difference in risk for retear between PRP-treated patients and those treated with arthroscopic repair alone. The total sample of patients was satisfying, as 1418 patients were examined in those studies. The risk ratio for retear was calculated to be 0.78 (*p* = 0.01), which shows a potential protective effect of PRP treatment for this quite common complication and possibly less need for revision surgical operations in the future although estimating the latter surpasses the goals of this review. It must be mentioned that the possible exception of the study by Dukan et al. [25] from the analysis (because of the difference in the definition of a retear incident to the other studies) does not have a significant impact on the outcome, as in this situation, the risk ratio was calculated to be 0.79 (*p* = 0.01).

### 3.7. Adverse Effects

Most of the trials reviewed reported either no adverse effects concerning the PRP treatment or similar rates of postoperative complications between treatment and control groups. However, Bergeson et al. [21] reported higher rate of infection in the PRFM group (12%) than in the control group (0%), as there were two cases of surgical site infection by Propionibacterium acnes. This difference did not reach statistical significance (*p* = 0.15). One case of infection by the same pathogen in the PRP group was reported by Flury et al. as well [26], but the difference of infection rates between groups was not statistically significant. These data are in accordance with the current literature, where PRP products are generally described as a safe treatment with scarce adverse effects.

## 4. Discussion

This review attempts to summarize the data available from clinical trials on the combination of arthroscopic repair and PRP treatment during the last years. Unfortunately, most of them recruited medium-to-small numbers of patients. As a result, in various cases, the authors have concluded that larger patient samples are needed. A quantitative analysis of the results already available from the literature is an attempt to help in this direction.

The most promising result of this review is the estimate that the risk for retear for the patients treated with PRP is about 22% lower than the risk for the patients under standard arthroscopic treatment. Insufficient data concerning the exact size and characteristics of the tears in the trials reviewed do not allow for a distinction between small and larger tears in this analysis. Other authors reviewing such trials have reached similar results. In a recent meta-analysis of level 1 studies, Chen et al. found that long-term retear rates are improved after PRP administration in rotator cuff tears [18]. In a systematic review of meta-analyses from 2016, Saltzman et al. [56] proposed that the use of PRP in the treatment of rotator cuff tear under specific variables, such as is the use of a solid PRP matrix, the application of PRP at the tendon–bone interface, in double-row repairs, and with small- and/or medium-sized rotator cuff tears, trends towards lower retear rates. However, they did not confirm a universal improvement of these rates.

Concerning the clinical results analyzed in this review, it is highly doubtful that PRP administration has had a significant impact on the postoperative rehabilitation of the patients. Although some statistically significant improvement in postoperative clinical scores (Constant–Murley, UCLA, and SST) was noted, this improvement is clearly lower than the respective MCID calculated in the literature available to the authors of this article. As a result, it cannot be proven that this improvement had an important effect on the pain, function, and activity levels of the patients. Similar results were reported by Xu et al. in a recent meta-analysis [57], after a review of 14 randomized controlled studies. According to this study, the postoperative Constant–Murley score has had a statistically significant improvement for the PRP-treated patients with large or massive tears but was lower than the MCID, and the UCLA score improved significantly for this subgroup as well but was close to the estimated threshold for the MCID, while the ASES scores had no statistically significant difference between PRP-treated patients and control groups. One should also not forget that PRP treatment poses an additional cost of the overall therapy of the patients. In a meta-analysis and cost-effectiveness analysis by Vavken et al. [58], it was estimated that although PRP treatment may reduce retear rates of small- and medium-sized tears, its use is not cost-effective concerning its clinical benefits. As this analysis refers to the costs and prices in USA in 2013, this aspect needs to be taken into account by future studies in order to determine the clinical value of such treatments.

The reasons for the difference between clinical scores and imaging results can be several. Successful rotator cuff surgery as measured by pain relief, functional recovery, and various outcome measures does not always require complete tendon healing [59]. The evaluation of clinical outcomes depends on the patient’s functional demands and subjective assessment to a certain degree. Patients with low functional demands and/or a lower activity level may benefit from rotator cuff repair despite a lack of complete healing and may also report acceptable functional results and higher satisfaction [60]. In a similar fashion, partial repair of massive rotator cuff tears can also yield outcomes comparable to those of complete repair of massive tears [61]. One should also keep in mind that intact tendon morphological features on imaging do not necessarily reflect tendon histologic characteristics and biochemical aspects [62].

We realize that this review has certain weaknesses, which have to be taken into account while evaluating the results. Firstly, the trials present the administration of different PRP products, as mentioned above. It is under discussion whether gel-like fibrin or liquid plasma has superior results as well as whether leukocyte-poor or leukocyte-rich PRP is preferable. As noted by Barber [63], while platelets can increase anabolic signaling, leukocytes increase catabolic signaling. The reason for the latter is that leukocyte-rich PRP demonstrates more matrix metalloproteinase-9 and 1L-1β (inflammatory catabolic mediators), which may be detrimental to tendon healing. Greater inflammatory responses were reported 5 days after treatment with leukocyte-rich PRP compared with leukocyte-poor PRP [64]. In addition, preparations with high leukocyte counts have also been implicated in poorer results [58]. The consequence was greater early tendon architecture disruption, higher vascularity, and fibrosis. Consequently, leukocyte-poor PRP may offer better healing. On the other hand, the studies analyzed by Chen [18] showed improved results in the Constant–Murley score for leukocyte-rich PRP, with otherwise no statistical differences between the results after the administration of leukocyte-poor and -rich PRP. In addition, the use of different PRP products and the lack of sufficient data concerning the exact composition of the biological treatment administered in most of the studies raise uncertainty of the true homogeneity of the mixtures administered, even among those with supposedly comparable composition and texture.

Secondly, another weakness is that there are differences of the surgical techniques among the trials reviewed. As mentioned above, it is still under discussion whether the supposedly better biomechanical construct of a double-row repair translates to better clinical results. It is, however, notable that better tendon healing shown in imaging studies and lower retear rates are expected after double-row studies, which are expected to result in lower revision rates [65,66]. Improvements upon the double-row repair have been attempted with the suture bridge repair, which isolates the healing area, avoiding flowing synovial fluids, and is estimated to be biomechanically superior regarding load resistance, pressure enhancement at the bone–tendon interface, and an increased coverage area [67]. Furthermore, the rehabilitation programs followed in each trial, although similar in many cases, were not identical. Differences in the postoperative protocols may be of major clinical importance, as physical therapy has shown to contribute to the alleviation of symptoms of rotator cuff tear even without surgical treatment [68].

In addition, it has to be noted that not all of the clinical trials assessed in this study are randomized clinical trials with a level I of evidence. The choice of inclusion trials of evidence level as low as three was made in order to be able to assess a wide range of trials and attempt statistical analysis of a bigger patient pool. However, we realize that further research with trials with stricter protocols needs to be undertaken in the future in order to further examine the above-mentioned results.

## 5. Conclusions

Our review has shown that the combination of PRP treatment and arthroscopic shoulder rotator cuff repair shows lower retear rates than arthroscopic repair alone, according to the analysis of the trials reviewed. Concerning the postoperative clinical scores, the statistically significant improvement of Constant–Murley, UCLA, and SST scores is lower than the minimal clinically important difference, and it is therefore uncertain whether they represent a higher level of functional and activity level in the patients’ everyday life. The lack of data concerning the exact composition of PRP products used and the different surgical techniques are weaknesses of the present study. There is a need for further studies in order to bolster and confirm the above results as well as to evaluate the cost-effectiveness and the ideal therapeutic scheme of such treatments.

## Figures and Tables

**Table 1:** Risk-of-bias assessment for randomized studies according to the Cochrane collaboration’s tool.

	Selection Bias		Performance Bias	Detection Bias	Attrition Bias	Reporting Bias	Other Bias
	Random sequence generation	Allocation concealment	Blinding of participants and researchers	Blinding of outcome assessment	Incomplete outcome data	Selective reporting	
Castricini et al. [10]	+	+	+	+	+	+	+
Randelli et al. [38]	+	+	+	+	+	+	+
Marquez et al. [36]	+	?	-	-	+	+	+
Gumina et al. [27]	+	+	+	+	+	+	+
Rodeo et al. [39]	+	+	+	-	+	+	?
Weber et al. [42]	+	+	+	+	+	+	+
Antuna et al. [19]	+	+	-	+	+	+	+
Jo et al. [31]	+	?	-	-	+	+	+
Malavolta et al. [33,34]	?	?	+	+	+	+	+
Jo et al. [32]	+	?	-	?	+	+	+
Ebert, Wang et al. [5,41]	+	?	-	-	+	+	+
Flury et al. [26]	+	+	+	+	+	+	+
Pandey et al. [37]	+	+	+	+	+	+	+
D’Ambrosi et al. [24]	+	+	?	+	+	+	+
Zumstein et al. [44]	+	?	+	+	+	+	+
Snow et al. [40]	+	+	+	+	+	+	+

Legend: low risk of bias, +; high risk of bias, -; unclear risk of bias, ?.

**Table 2:** Assessment of non-randomized studies according to the Newcastle–Ottawa quality assessment scale for cohort studies.

	Selection				Comparability	Outcome			Total
	Representativeness of the exposed cohort	Selection of the non-exposed cohort	Ascertainment of exposure	Demonstration that outcome of interest was not present at start	Comparability of cohorts on the basis of the design or analysis	Assessment of outcome	Was follow-up long enough for outcomes to occur	Adequacy of follow up of cohorts	
Barber et al. [20]	⁎	⁎	⁎	⁎	⁎⁎	⁎	⁎	⁎	9
Jo et al. [30]	⁎	⁎	⁎	⁎	⁎⁎	⁎	⁎	⁎	9
Bergeson et al. [21]	⁎	⁎	⁎	⁎	⁎⁎	⁎	⁎	⁎	9
Buford [46]			⁎	⁎	⁎⁎	⁎	⁎	⁎	7
Charousset et al. [23]	⁎	⁎	⁎	⁎	⁎⁎	⁎	⁎		8
Zhang et al. [43]	⁎	⁎	⁎	⁎	⁎⁎	⁎	⁎	⁎	9
Gwinner et al. [28]	⁎	⁎	⁎	⁎	⁎⁎	⁎	⁎	⁎	9
Dukan et al. [25]	⁎	⁎	⁎	⁎	⁎⁎	⁎	⁎	⁎	9
Auregan et al. [45]	⁎	⁎	⁎	⁎	⁎⁎	⁎			7

Legend: ⁎, ⁎⁎: number of “stars” allocated according to the Newcastle–Ottawa scale.

**Table 3:** Comparison of post-operative Constant–Murley scores between PRP and control group at final follow-up.

	PRP + Arthroscopy			Arthroscopy				Mean Difference
Study	Mean	SD	Total	Mean	SD	Total	Weight	IV, Fixed, 95% CI
Auregan et al. [45]	77	13.5	26	72.4	12.3	23	2.2%	4.6 [−2.62, 11.82]
Castricini et al. [10]	88.4	7.62	43	88.4	7.78	45	10.8%	0 [−3.22, 3.22]
Charousset et al. [23]	77.3	9.9	31	78.1	7.7	30	5.7%	−0.8 [−5.24, 3.64]
D’Ambrosi et al. [24]	81	11.2	20	78.5	9	20	2.8%	2.5 [−3.8, 8.8]
Dukan et al. [25]	86.7	11.1	32	81.6	14.4	37	3.1%	5.1 [−0.93, 11.13]
Ebert, Wang et al. [5,41]	86.2	11.4	27	85.2	11.3	28	3.1%	1.0 [−5.0, 7.0]
Flury et al. [26]	82.7	8	49	82.1	9.5	52	9.6%	0.6 [−2.82, 4.02]
Gumina et al. [27]	77.9	5.7	39	74.2	6.1	37	15.9%	3.7 [1.04, 6.36]
Gwinner et al. [28]	79	13	18	77	13	18	1.6%	2.0 [−6.49, 10.49]
Jo et al. [30]	79.12	13.42	19	82	13.02	23	1.7%	−2.88 [−10.93, 5.17]
Jo et al. [31]	74.82	14.3	24	69.84	16.29	24	1.5%	4.98 [−3.69, 13.65]
Jo et al. [32]	74.67	9.17	37	70.87	9.76	37	6%	3.8 [−0.52, 8.12]
Malavolta et al. [33,34]	82.1	11	26	82	9.5	25	3.5%	0.1 [−5.53, 5.73]
Marquez et al. [36]	65.6	13.1	14	64.1	13.6	14	1.1%	1.5 [−8.39, 11.39]
Pandey et al. [37]	93.2	4.97	52	87.6	8.12	50	16.3%	5.6 [2.98, 8.22]
Randelli et al. [38]	82.4	6.3	22	78.7	10	23	4.8%	3.7 [−1.16, 8.56]
Snow et al. [40]	72.8	19.36	40	72.6	18.54	47	1.8%	0.2 [−7.81, 8.21]
Zhang et al. [43]	81.5	7.7	30	80.3	6.7	30	8.4%	1.2 [−2.45, 4.85]
Total (95% CI)			549			563	100%	2.46 [1.4, 3.52]

Heterogeneity: Chi² = 17.21, df = 17 (*p* = 0.44), I² = 1%. Test for Overall Effect: Z = 4.56 (*p* < 0.00001).

**Table 4:** Comparison of post-operative ASES scores between PRP and control groups at final follow-up.

	PRP + Arthroscopy			Arthroscopy				Mean Difference
Study	Mean	SD	Total	Mean	SD	Total	Weight	IV, Fixed, 95% CI
Flury et al. [26]	92	12	50	92.5	12.8	54	12.2%	−0.5 [−5.27, 4.27]
Jo et al. [30]	87.61	24.83	19	89.92	17.03	23	1.6%	−2.31 [−15.47, 10.85]
Jo et al. [31]	88.94	13.61	24	85.56	17.26	24	3.6%	3.38 [−5.41, 12.17]
Jo et al. [32]	87.96	13.1	37	83.65	14.56	37	7%	4.31 [−2.0, 10.62]
Pandey et al. [37]	87.9	5.73	52	86.1	6.2	50	51.7%	1.8 [−0.52, 4.12]
Rodeo et al. [39]	91.3	9.53	19	96.43	5.55	22	11.7%	−5.13 [−10.0, −0.26]
Snow et al. [40]	80.1	21.46	40	74.2	25.36	47	2.9%	5.9 [−3.94, 15.74]
Weber et al. [42]	82.48	8.77	29	82.52	12.45	30	9.3%	−0.04 [−5.52, 5.44]
Total (95% CI)			270			287	100%	0.82 [−0.85, 2.49]

Heterogeneity: Chi² = 9.55, df = 7 (*p* = 0.22), I² = 27. Test for overall effect: Z = 0.96 (*p* = 0.34).

**Table 5:** Comparison of post-operative SST scores between PRP and control groups at final follow-up.

	PRP + Arthroscopy			Arthroscopy				Mean Difference
Study	Mean	SD	Total	Mean	SD	Total	Weight	IV, Fixed, 95% CI
Charousset et al. [23]	9.9	2.9	35	10.2	2	35	6.9%	−0.3 [−1.47, 0.87]
Gumina et al. [27]	10.5	0.8	39	10.1	1	37	56%	0.4 [−0.01, 0.81]
Jo et al. [30]	9.83	3.31	19	10.57	1.73	23	3.4%	−0.74 [−2.39, 0.91]
Jo et al. [31]	10.33	2.3	24	9.88	2.79	24	4.5%	0.45 [−1.0, 1.9]
Jo et al. [32]	10.24	2.14	37	9.76	2.27	37	9.3%	0.48 [−0.53, 1.49]
Randelli et al. [38]	11.3	0.9	22	10.9	1.4	23	19.9%	0.4 [−0.28, 1.08]
Total (95% CI)			176			179	100%	0.32 [0.02, 0.63]

Heterogeneity: Chi² = 3.0, df = 5 (*p* = 0.7), I² = 0. Test for overall effect: Z = 2.07 (*p* = 0.04).

**Table 6:** Comparison of post-operative UCLA scores between PRP and control groups at final follow-up.

	PRP + Arthroscopy			Arthroscopy				Mean Difference
Study	Mean	SD	Total	Mean	SD	Total	Weight	IV, Fixed, 95% CI
Charousset et al. [23]	29.1	2.3	35	30.3	3.2	35	21.2%	−1.2 [−2.51, 0.11]
Jo et al. [30]	31.78	6.15	19	30.83	4.96	23	3.1%	0.95 [−2.48, 4.38]
Jo et al. [31]	30.13	3.98	24	29.21	6.04	24	4.3%	0.92 [−1.97, 3.81]
Jo et al. [32]	30.73	4.15	37	29.54	4.86	37	8.5%	1.19 [−0.87, 3.25]
Malavolta et al. [33,34]	32.1	4.6	26	32.5	3.8	25	6.8%	−0.4 [−2.71, 1.91]
Pandey et al. [37]	34.75	0.72	52	32.22	3.55	50	35.9%	2.53 [1.53, 3.53]
Randelli et al. [38]	33.3	2.2	22	31.3	4.1	23	9.9%	2.0 [0.09, 3.91]
Weber et al. [42]	27.94	4.98	30	29.59	1.68	30	10.2%	−1.65 [−3.53, 0.23]
Total (95% CI)			245			247	100%	0.83 [0.23, 1.43]

Heterogeneity: Chi² = 29.65, df = 7 (*p* = 0.0001), I² = 76%. Test for overall effect: Z = 2.7 (*p* = 0.007).

**Table 7:** Comparison of post-operative retear rates between PRP and control groups at final follow-up.

	Arthroscopy + PRP		Arthroscopy			Risk Ratio
Study	Retear incidents	Patients number	Retear incidents	Patients number	Weight	M-H, Fixed, 95% CI
Antuna et al. [19]	13	14	10	14	5.9%	1.3 [0.91, 1.87]
Auregan et al. [45]	10	26	7	23	4.4%	1.26 [0.58, 2.77]
Barber et al. [20]	6	20	12	20	7.1%	0.5 [0.23, 1.07]
Bergeson et al. [21]	9	16	8	21	4.1%	1.48 [0.74, 2.96]
Buford [46]	2	50	3	50	1.8%	0.67 [0.12, 3.82]
Castricini et al. [10]	1	40	4	38	2.4%	0.24 [0.03, 2.03]
Charousset et al. [23]	11	31	12	30	7.2%	0.89 [0.46, 1.69]
D’Ambrosi et al. [24]	0	20	0	20		Not estimable
Dukan et al. [25]	3	32	5	37	2.7%	0.69 [0.18, 2.68]
Ebert, Wang et al. [5,41]	2	29	3	30	1.7%	0.69 [0.12, 3.83]
Flury et al. [26]	5	49	9	53	5.1%	0.6 [0.22, 1.67]
Gumina et al. [27]	0	39	3	37	2.1%	0.14 [0.01, 2.54]
Gwinner et al. [28]	2	18	5	18	2.9%	0.4 [0.09, 1.8]
Jo et al. [30]	4	15	7	17	3.9%	0.65 [0.24, 1.78]
Jo et al. [31]	4	20	10	18	6.2%	0.36 [0.14, 0.95]
Jo et al. [32]	1	33	6	30	3.7%	0.15 [0.02, 1.19]
Malavolta et al. [33,34]	0	22	1	22	0.9%	0.33 [0.01, 7.76]
Marquez et al. [36]	9	14	6	14	3.5%	1.5 [0.73, 3.08]
Pandey et al. [37]	2	52	10	50	6.0%	0.19 [0.04, 0.83]
Randelli et al. [38]	9	22	12	23	6.9%	0.78 [0.41, 1.48]
Rodeo et al. [39]	12	36	6	31	3.8%	1.72 [0.73, 4.05]
Snow et al. [40]	6	39	8	38	4.8%	0.73 [0.28, 1.91]
Weber et al. [42]	12	28	7	24	4.4%	1.47 [0.69, 3.13]
Zhang et al. [43]	4	30	9	30	5.3%	0.44 [0.15, 1.29]
Zumstein et al. [44]	6	17	6	18	3.4%	1.06 [0.42, 2.65]
Total (95% CI)	133	712	169	706	100%	0.78 [0.65, 0.94]

Heterogeneity: Chi² = 36.76%, df = 23 (*p* = 0.03), I² = 37%. Test for overall effect: Z = 2.58, (*p* = 0.01).

## Data Availability

Not applicable.
